# The reduced SCFA-producing gut microbes are involved in the inflammatory activation in Kawasaki disease

**DOI:** 10.3389/fimmu.2023.1124118

**Published:** 2023-06-15

**Authors:** Fangyan Wang, Fanyu Qian, Qihao Zhang, Jian Zhao, Jianke Cen, Jiamin Zhang, Jinhui Zhou, Ming Luo, Chang Jia, Xing Rong, Maoping Chu

**Affiliations:** ^1^ Department of Pathophysiology, School of Basic Medical Science, Wenzhou Medical University, Wenzhou, China; ^2^ The Research Institute of Microbiota and Host Inflammation-Related Diseases, Wenzhou Medical University, Wenzhou, China; ^3^ Pediatric Research Institute, The Second Affiliated Hospital and Yuying Children’s Hospital of Wenzhou Medical University, Wenzhou, China; ^4^ Department of Reproductive Endocrinology, Women’s Hospital, School of Medicine, Zhejiang University, Hangzhou, China; ^5^ Key Laboratory of Structural Malformations in Children of Zhejiang Province, Wenzhou, China; ^6^ School of Ophthalmology and Optometry, Wenzhou Medical University, Wenzhou, China; ^7^ Children’s Heart Center, Institute of Cardiovascular Development and Translational Medicine, The Second Affiliated Hospital and Yuying Children’s Hospital of Wenzhou Medical University, Wenzhou, China

**Keywords:** Kawasaki disease, gut microbiota, butyrate, SCFAs, MAPK, macrophage

## Abstract

Kawasaki disease (KD), an acute febrile systemic vasculitis in children, has become the leading cause of acquired heart disease in developed countries. Recently, the altered gut microbiota was found in KD patients during the acute phase. However, little is known about its characteristics and role in the pathogenesis of KD. In our study, an altered gut microbiota composition featured by the reduction in SCFAs-producing bacteria was demonstrated in the KD mouse model. Next, probiotic *Clostridium butyricum* (*C. butyricum*) and antibiotic cocktails were respectively employed to modulate gut microbiota. The use of *C. butyricum* significantly increased the abundance of SCFAs-producing bacteria and attenuated the coronary lesions with reduced inflammatory markers IL-1β and IL-6, but antibiotics depleting gut bacteria oppositely deteriorated the inflammation response. The gut leakage induced by dysbiosis to deteriorate the host’s inflammation was confirmed by the decreased intestinal barrier proteins Claudin-1, Jam-1, Occludin, and ZO-1, and increased plasma D-lactate level in KD mice. Mechanistically, SCFAs, the major beneficial metabolites of gut microbes to maintain the intestinal barrier integrity and inhibit inflammation, was also found decreased, especially butyrate, acetate and propionate, in KD mice by gas chromatography-mass spectrometry (GC-MS). Moreover, the reduced expression of SCFAs transporters, monocarboxylate transporter 1 (MCT-1) and sodium-dependent monocarboxylate transporter 1 (SMCT-1), was also shown in KD mice by western blot and RT-qPCR analyses. As expected, the decrease of fecal SCFAs production and barrier dysfunction were improved by oral *C. butyricum* treatment but was deteriorated by antibiotics. *In vitro*, butyrate, not acetate or propionate, increased the expression of phosphatase MKP-1 to dephosphorylate activated JNK, ERK1/2 and p38 MAPK against excessive inflammation in RAW264.7 macrophages. It suggests a new insight into probiotics and their metabolites supplements to treat KD.

## Introduction

Kawasaki disease (KD), an acute febrile systemic vasculitis of small- to medium-sized blood vessels in children, has become the major cause of acquired heart disease in developed countries (1, 2). Coronary artery aneurysms are the most severe cardiovascular complication of KD, leading to important morbidity and mortality. Despite insufficient knowledge of its etiology and pathophysiology, excessive inflammatory response was suggested in reference to activated macrophages with increased cytokines, adhesion molecules and chemokines during vascular inflammation (1).

After intestinal colonization of some environmental bacteria at birth, gut microbiota gradually evolves to constitute a relatively stable pattern at the preschooler stage, which is vulnerable to food intake and reciprocally interacts with host healthy conditions (3, 4). Increasing findings showed dysbiosis as well as gut leakage and microbial metabolite alterations were involved in pediatric diseases. Interestingly, it was estimated that over 60% of KD patients developed gastrointestinal symptoms, including abdominal pain, vomiting, diarrhea or constipation during the acute phase (5, 6). Recently, the altered gut dysbiosis with the proliferation of *Streptococcus*, *Enterococcus*, and decreased abundance of *Lactobacillus* (7–9) was shown during the febrile phase of KD patients. However, the detailed role of gut microbiota in KD remains unclear.

Short-chain fatty acids (SCFAs) are well-known metabolites of gut microbiota to interact with the hosts (10). Typically, among SCFAs, the bioactivities of acetate, propionate and butyrate have been extensively reported. SCFAs can provide 70% energy fuel for the intestinal epithelial cells to maintain the integrity of intestinal barrier (11). Published studies showed the leaky gut with intestinal barrier dysfunction causing the increase of pathogen-associated molecular patterns (PAMP) in blood to further deteriorate inflammatory response was attributed to the reduction of fecal SCFAs (11, 12). Besides, SCFAs can serve to modulate the leukocytes-induced inflammation in different diseases through their special receptor GPR41, 43 and 109A (13, 14). For butyrate, it was reported as the histone deacetylase inhibitor to dramatically inhibit excessive inflammation in septic mice (15). However, whether the gut microbiota in KD could act on the inflammation *via* SCFAs in KD needs to be clarified.

In this study, we established the KD mouse model using *C. albicans* water-soluble fraction (CAWS) injection to investigate the altered gut microbiota pattern, intestinal barrier proteins and coronary inflammatory lesions for the evaluation of gut microbiota role in KD. Furthermore, the microbial metabolites SCFAs were targeted for molecular mechanism research in the cultured RAW264.7 macrophages.

## Materials and methods

### Ethics

All animal experiments complied with the Guide for the Care and Use of Laboratory Animals in China and were approved by the Animal Care and Use Committee of Wenzhou Medical University (wydw 2017-0046).

### Preparation of CAWS

According to previous studies (16, 17), we cultivated *Candida albicans* strain NBRC1385 in the C-limiting medium (270 rpm, 27°C, 48h). Then, an equivalent volume of ethanol was added to the medium and the mixture was kept at 4°C overnight. Next, we centrifuged the cultures and collected the pellets. Dissolve the pellets with water and centrifuge again to obtain the soluble part. Add an equivalent volume of ethanol before being kept at 4°C overnight. Repeat centrifugation to collect the pellets. Last, we added acetone to refine the pellets and obtained the final CAWS product when the acetone vaporized. At the time of preparing the CAWS solution, we dissolved the powder with phosphate-buffered saline (PBS) and it will be autoclaved before use.

### Grouping and KD modeling

C57BL/6J WT male mice at the age of 3-4 weeks were randomly divided into four groups (n=10 for each group): PBS group, CAWS group, and CAWS + *C. butyricum* group, CAWS + antibiotics (ABX) group. For the CAWS-treated group, mice were injected intraperitoneally with 4mg/kg of CAWS solution for 5 days. The PBS group was equally injected intraperitoneally with PBS buffer. *C. butyricum* was prepared for daily oral administration (5×10^6^ CFU/g) that started from the first day of CAWS modeling and lasted for 4 weeks. In the CAWS + ABX group, mice were treated with 5-day antibiotic cocktails of 1mg/ml streptomycin, neomycin, and bacitracin dissolved in PBS at the time of the CAWS modeling. In the addition experiment of butyrate, we added 100mM sodium butyrate in the daily drinking water for mice since the day of CAWS modeling. After 4 weeks of the final CAWS injection, mice were anesthetized and sacrificed for the heart and colon tissues.

### Gut microbiota analysis

Mice fecal samples were collected and frozen at −80°C for storage. We used the 16S ribosomal ribonucleic acid (16S rRNA) sequencing to detect the V3 and V4 regions. The 16S rRNA community profile was evaluated by Novogene’s Illumina MiSeq Technology (Suzhou, China) and analyzed on NovoMagic (https://magic.novogene.com/) to assign operational taxonomic units (OTUs). Representative sequences of each OTU were used to examine the taxonomy assignment at different levels. We generated the principal component analysis (PCA) plots for visualization of UniFrac dissimilarity using the QIIME pipeline. The characteristics of differences were assessed through linear discriminant analysis effect size (LEfSe).

### Gas chromatography-mass spectrometry quantification of short-chain fatty acids

Stool samples were homogenized in phosphoric acid and isohexanoic acid solution at 70 Hz and centrifuged at 4 °C to collect the supernatant for GC-MS analysis. The GC was fitted with a capillary column Agilent HP-INNOWAX (Agilent Technologies, Santa Clara, CA, USA). The temperature was initially set at 90 °C, then was increased to 120 °C at 10 °C/min, to 150 °C at 5 °C/min, and finally to 250 °C at 25 °C/min and kept at this temperature for 2 min (total running time:15min). The detector was operated using the electron impact ionization mode (electron energy: 70 eV), the full scan mode, and the selective ion monitoring (SIM) mode.

### Hematoxylin and eosin staining

Colon and heart tissues were fixed in 4% paraformaldehyde and embedded with paraffin. Five-µm-thick sections of tissues were dewaxed and hydrated and then stained with H&E solution. All slides were observed under a light microscope and images were obtained using an SCN400 slide scanner and Digital Image Hub software (Nikon, Tokyo, Japan).

### Immunofluorescence

The colon and heart tissues were de-paraffinized and rehydrated before incubation within 3% H_2_O_2_ and 5% BSA for the block. Afterwards, the heart sections were incubated with primary antibodies against CD31(Abcam, ab24950, 1:1000), ICAM-1 (Affinity Biosciences. AF6088, 1:100) and CD68 (Proteintech, 28058-1-AP, 1:100), and primary antibodies against Claudin-1 (Proteintech, 13050-1-AP, 1:100), Jam-1 (Affinity Biosciences. DF6373, 1:100), Occludin (Proteintech, 27260-1-AP, 1:100), ZO-1 (Proteintech, 21773-1-AP, 1:300), MCT-1 (Proteintech, 20139-1-AP, 1:50) and SMCT-1 (SLC5A8, Proteintech, 21433-1-AP, 1:50) were used for colon sections. LFA-1 (ITGAL, Affinity Biosciences. DF5625, 1:50) was used for the RAW264.7. Next, sections were washed and cultured with fluorescent secondary antibodies. Images were examined under a laser scanning confocal microscope (Nikon, A1 PLUS, Tokyo, Japan).

### Cells culture and treatment

The mouse monocytic leukemia cell line RAW264.7 (American Type Culture Collection, Manassas, VA, USA) was plated in 6-,12-well plates (Becton Dickinson Lab-ware, Franklin Lakes, NJ) and cultured in high glucose Dulbecco’s modified Eagle’s medium (DMEM) supplemented with 10% fetal bovine serum (FBS) at 37°C with 5% CO_2_/95% air. No mycoplasma contamination was found. CAWS (0.5mg/ml) or an equivalent volume of PBS was added into the medium and treated RAW264.7 cells for 24h. Butyrate (1mM) was used an hour before CAWS administration.

### Quantitative reverse transcription-polymerase chain reaction

Tissues and cells were obtained and lysed in TRIzol. Markers for RT-qPCR were selected: Claudin-1, Occludin-1, Jam-1, ZO-1, MCT-1, SMCT-1, IL-1β, IL-6, IL-8, TNF-α, LFA-1, MCP-1, MKP-1, PP1, PP2, PP2A, PTP1B and SHP2. All mRNA samples were extracted and transcribed into cDNA according to standard protocols (TAKARA, PrimeScript RT Master Mix (Perfect Real Time), RR036A). 5 ng/µL cDNA was prepared with 1.6 µL each of forward and reverse primers (5µM) and 10 µL SYBR Green PCR Master Mix (TAKARA, TB GreenTM Premix Ex TaqTM II (Tli RNaseH Plus, RR820A)) before performing on the LightCycler R 480 Real-Time PCR System (Roche Molecular Systems, Inc. Indiana, USA). The expression of all the target genes was normalized to GAPDH. The relative changes in gene expressions were calculated using the 2^−ΔΔCt^ method. The primers used in the study were shown in **Supplementary Table 1**.

### Western blot analysis

Total proteins were extracted from heart tissues or RAW264.7 cell lysates and supplemented with a cocktail of protease inhibitors and phosphatase inhibitors. Proteins were quantified to an equivalent amount, separated by SDS-PAGE gel, and electro-transferred to a PVDF membrane. Having been blocked in 5% skimmed milk, membranes were incubated with antibodies: anti-LFA-1 (ITGAL, Affinity Biosciences. DF5625, 1:1000), anti-p-JNK (Affinity Biosciences. AF3318, 1:1000), anti-total JNK (Affinity Biosciences. AF6138, 1:1000), anti-p-ERK (Affinity Biosciences. AF1015, 1:1000), anti-total ERK (Affinity Biosciences. AF0155, 1:1000), anti-p-p38(Affinity Biosciences. AF4001, 1:1000), anti-total p38 (Affinity Biosciences. AF6456, 1:1000) and GAPDH. The loading control was GAPDH (Proteintech, 60004-1-lg, 1:10000). Western blot bands were analyzed with the ChemiDicTM XRS + Imaging System (Bio-Rad Laboratories, Hercules, CA, USA), and the densities were quantified with Multi Gauge Software of Science Lab 2006 (FUJIFILM Corporation, Tokyo, Japan).

### Enzyme-linked immunosorbent assay

Concentrations of IL-1β, IL-6, TNF-α, MCP-1, and D-lactate in the plasma were determined by the corresponding ELISA kits (Shanghai Westang Bio-Tech CO.LTD, Shanghai, China) following the standard protocols. For examining the concentration of inflammatory cytokines in RAW264.7 cell supernatants, RAW264.7 cells were stimulated with CAWS and then washed with PBS for 24h. After that, the levels of inflammatory cytokines were determined following the manufacturer’s instructions.

### Statistical analysis

All data were analyzed using SPSS version 19.0 (IBM, Armonk, NY, USA). Tests of normality were performed and data were normally distributed. A two-tailed unpaired Student’s t-test was used to compare two experimental groups, and one-way analysis of variance (ANOVA) followed by Duncan’s multiple-range test was performed for the comparison among more than two groups. Values are expressed as the mean ± SEM.

## Results

### Gut dysbiosis was involved in the coronary artery inflammatory injury of KD mice

**Figure 1:**
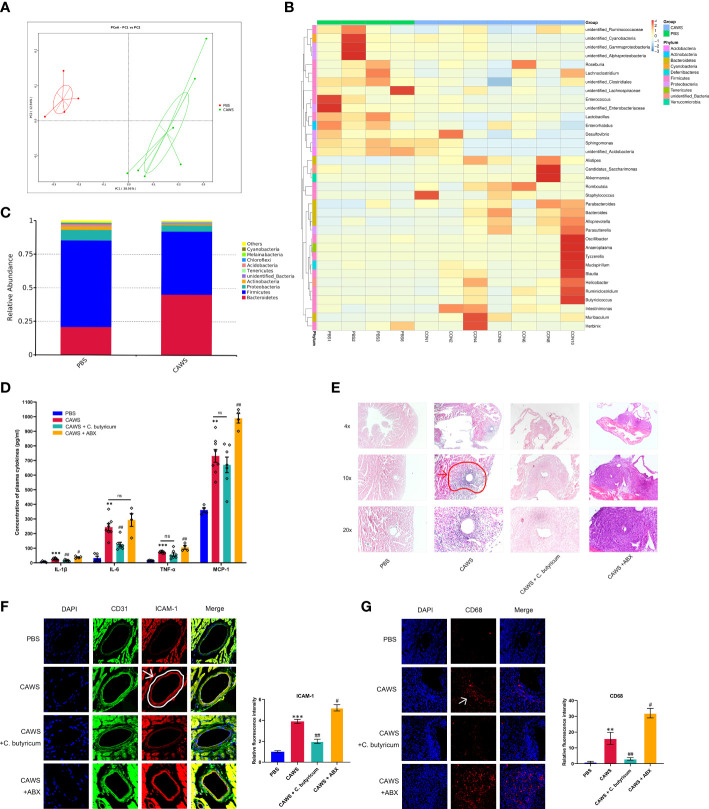
Gut dysbiosis played a role in the inflammation and coronary artery injury of the KD mouse model. **(A)** PCoA plots of bray-curtis similarity matrices between PBS group (n=4) and CAWS group (n=7). **(B)** Heatmap of gut microbiota on the genus level. **(C)** The relative abundance of the gut microbiota in phylum level. **(D)** Plasma cytokine levels of IL-1β, IL-6, TNF-α, MCP-1. **(E)** H&E staining of heart tissues. The coronary artery lesion in the CAWS group was circled in red and pointed out by an arrow. Magnification: ×200. Scale bar = 20 μm. **(F, G)** The expression levels of intercellular cell adhesion molecule-1 (ICAM-1) and macrophage marker CD68 were evaluated using immunofluorescence of coronary arteries. Magnification: × 200. Scale bars = 20 µm. Values are expressed as the mean ± SEM. Significance: ** P < 0.01, *** P < 0.001, *vs.* the PBS group; ^#^ P < 0.05, ^##^ P < 0.01 *vs.* the CAWS group; ns means no significance.

We observed significant changes in fecal microbiota compositions of the CAWS group using principal components analysis and abundance clustering heatmap (**Figures 1A, B**; **Supplementary Figures 1A, B**). In the CAWS group, the relative abundance of *Bacteroidetes* was approximately twice as much as that in the PBS group while *Firmicutes* was dwindled by one third. Moreover, another manifested feature of gut microbiota in CAWS group was the decrease of *Proteobacteria* and *Actinobacteria* on phylum level (**Figure 1C**). **Figures 1D, E** show increased level of plasma inflammatory markers, IL-1β, IL-6, TNF-α, and monocyte chemoattractant protein-1 (MCP-1) and aggravated severity of coronary lesions in CAWS group. Interestingly, when using probiotic *C. butyricum*, the decreased IL-1β and IL-6 level in the plasma were observed with the attenuated vascular lesions, compared to more severe coronary lesions and increased chemokine MCP-1 in the CAWS+ABX group. Moreover, we found increased expression of the adhesion molecules CD31, ICAM-1 and CD68, indicating the activated neutrophil and macrophages recruitment and infiltration in the coronary artery wall (**Figures 1F, G**). Different gut microbiota status was associated with inflammatory conditions, manifested by the less relative fluorescence intensity of adhesion molecules in CAWS + *C. butyricum* group while higher intensity in ABX group. Together, we supposed the gut dysbiosis plays a crucial role in inflammatory responses of the KD mouse model during the acute phase and the use of antibiotics may have a worsening effect.

### The SCFAs-producing bacteria was reduced in the KD mouse model

**Figure 2:**
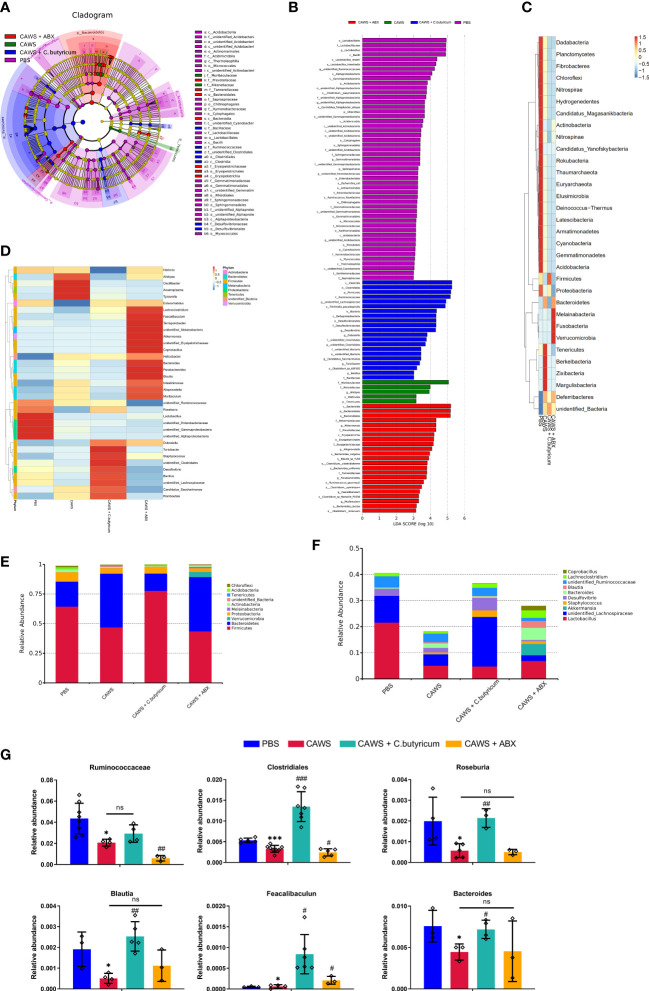
The SCFAs-producing bacteria were reduced in the KD mouse model. **(A, B)** The LEfSe analysis was employed to compare the taxonomic abundances. LDA=3. **(C, D)** Heat maps showed the alteration of relative abundance in phylum level **(C)** and genus level **(D)**. **(E, F)** The relative abundance of gut microbiota was portrayed in phylum level **(E)** and genus level **(F)**. **(G)** The relative abundance of several SCFAs-producing bacteria was shown. Values are expressed as the mean ± SEM. Significance: * P < 0.05, *** P < 0.001, vs. the PBS group; ^#^ P < 0.05, ^##^ P < 0.01, ^###^ P < 0.01 vs. the CAWS group; ns means no significance.

According to the LEfSe differential analysis, we identified 42 significant discriminative features (LDA>3) at the class (n=12), order (n=15) and family (n=16) levels (**Figures 2A, B**). *Lactobacillus* was the dominant phylum in the PBS group while *Muribaculaceae* and *Rikenellaceae* in the *Bacteroidetes* phylum were enriched in CAWS groups. Moreover, antibiotic use increased the abundance of *Bacteroidia* and some of the mucin-degrading family *Akkermansiaceae*, while the addition of probiotic *C. butyricum* significantly improved the population of *Firmicutes*. In **Figures 2C, D**, heat maps illustrated the differences in the relative abundance of phylum level and genus level. The heatmaps demonstrated that the gut microbial community was significantly changed due to the administration of CAWS, and microbial interventions further modulated the gut microbiota composition leading to different gut microbiota phenotypes. Next, we portrayed the relative abundance of gut microbiota at the phylum level (**Figure 2E**) and genus level (**Figure 2F**). Significantly, we observed a lower total abundance of gut microbiota at the genus level in the CAWS group, especially SCFAs-producing bacteria such as *Lactobacillus*. While with the treatment of *C. butyricum*, the increased total abundance and high abundance of *Firmicutes* implied the successful implantation and suggested its probiotic effect in promoting the gut microbial community. Interestingly, when the mice of CAWS group received the antibiotic treatment, the total abundance of gut microbiota came oppositely larger. It’s no surprise that the antibiotics will kill bacteria while potentially boosting the proliferation of harmful opportunistic pathogens and leaving more vacant niches for antibiotic-resistant bacteria (3). In CAWS + ABX group, *Lachnoclostridium* was detected increased in CAWS + ABX group, which could promote the production of trimethylamine (TMA) harmful to cardiovascular health and the abundance of some SCFAs-producing bacteria was detected decreased including *Ruminococcaceae* and *Lachnospiraceae*. Moreover, mice in the CAWS + ABX group were treated with 5-day antibiotic cocktails at the time of the CAWS modeling. Thus, before the final day when we collected the fecal samples, there have been some time interval for gut microbiota to restore, causing the increased abundance in CAWS + ABX group. In **Figure 2G**, it shows six significant SCFAs-producing gut microbes between the PBS group and CAWS group: *Ruminococcus*, *Clostridium*, *Roseburia*, *Blautia*, *Faecalibacteriu*, and *Bacteroides*, five of which were increased with the *C. butyricum* supplement. Taken together, we characterized the gut dysbiosis in CAWS group as the shrinking of the total gut microbiota community, particularly the decrease of SCFAs-producing bacteria which could thereby influence the inflammation and coronary artery injuries.

### Gut dysbiosis contributed to the leaky gut in the KD mouse model

**Figure 3:**
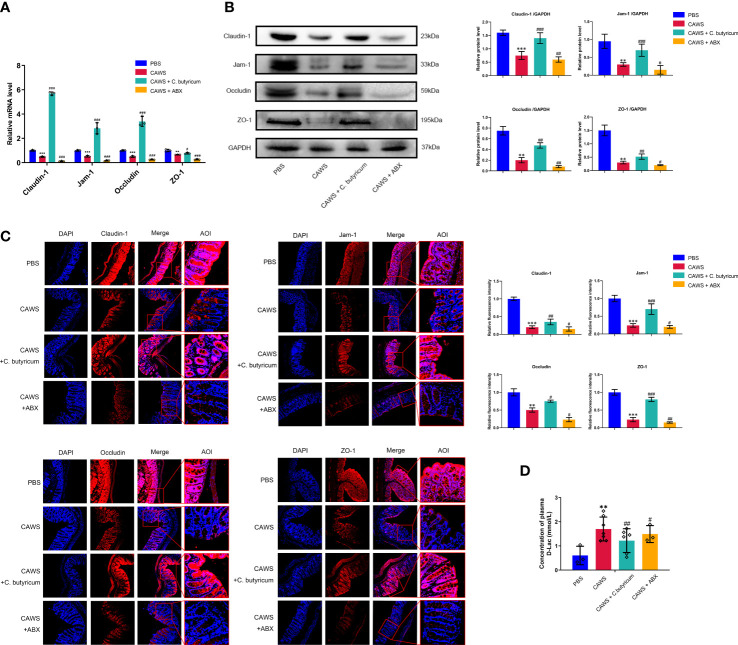
Gut dysbiosis contributed to the leaky gut in the KD mouse model. **(A, B)** The expression of intestinal barrier proteins Claudin-1, Jam-1, Occludin and ZO-1 was assessed by qPCR and western blot when using *C.butyricum* or antibiotics. **(C)** Immunofluorescence staining showed different expressions of intestinal barrier proteins among groups. Magnification: × 200. Scale bars = 100 µm. **(D)** The concentration of plasma D-Lac was detected in PBS group (n=3), CAWS group (n=7), CAWS+*C. butyricum* group (n=6) and CAWS+ABX group (n=3). Values are expressed as the mean ± SEM. Significance: ** P < 0.01, *** P < 0.001, vs. the PBS group; ^#^ P < 0.05, ^##^ P < 0.01, ^###^ P < 0.01 vs. the CAWS group; ns means no significance.

Since the excessive inflammatory response could consequently cause injury to tissues and organs, we aimed to evaluate the function of the gut barrier in the KD mouse model. According to **Figures 3A–C**, it exhibited decreased expression of tight junction proteins Claudin-1, Jam-1, Occludin, and ZO-1 in the KD mouse model. And with the probiotic supplement, we observed the restoration of tight junction proteins in the intestinal barrier, where the antibiotics depleting gut bacteria oppositely deteriorated its integrity. For evaluating the intestinal permeability, we measured the plasma level of D-lactate produced by the intestinal bacteria and physiologically limited in gut lumen. In **Figure 3D**, it showed higher level of D-lactate in the CAWS group than the PBS group, and interestingly, when *C. butyricum* was supplemented, the leaky gut condition was alleviated with decreased D-lactate levels. In this regard, these findings suggested the gut barrier impairment in KD mouse model to aggravate inflammation response of KD.

### The production and absorption of SCFAs were decreased for the shrink of SCFAs-producing bacteria community in the KD mouse model

**Figure 4:**
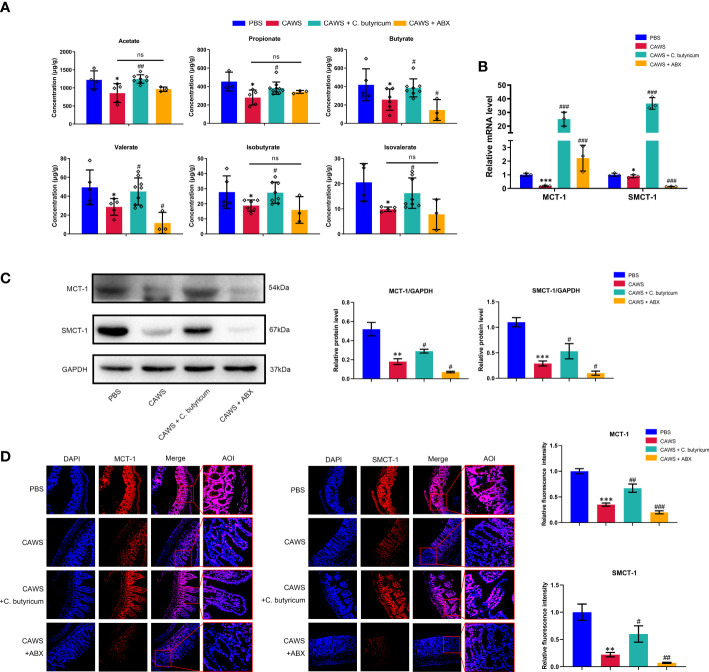
The production and absorption of SCFAs were decreased for the shrink of SCFAs-producing bacteria community in the KD mouse model. **(A)** Concentrations of SCFA acetate, propionate, butyrate, valerate, iso-butyrate and isovalerate were determined in fecal samples using GC-MS quantification. **(B, C)** The expression of monocarboxylic acid transporters MCT-1 and SMCT-1 in colon were evaluated using qPCR and western blot. **(D)** Relative fluorescence intensity was evaluated using immunofluorescent assay for monocarboxylic acid transporters. Magnification: × 200. Scale bars = 100 µm. Values are expressed as the mean ± SEM. Significance: * P < 0.05, ** P < 0.01, *** P < 0.001, vs. the PBS group; ^#^ P < 0.05, ^##^ P < 0.01, ^###^ P < 0.01 vs. the CAWS group; ns means no significance.

SCFAs are one of the most significant metabolites of gut microbes to maintain the intestinal barrier integrity and inhibit inflammation, especially butyrate, acetate and propionate. Presumably, the shrink of SCFAs production and decreased absorption could undermine their protective effects to contribute the inflammation of KD. In our study, we first detected the reduction in the SCFAs-producing bacteria by 16S rRNA sequencing and then employed gas chromatography-mass spectrometry (GC-MS) quantification to measure fecal SCFAs level, including acetate, propionate, butyrate, valerate, isobutyrate, and isovalerate. In **Figure 4A**, it shows a notable decrease in all detected SCFAs in the CAWS group in line with the decreased abundance of SCFAs-producing bacteria. The transportation of SCFAs into the circulation is facilitated by their transporters, mainly MCT-1 and SMCT-1 (18). Going through **Figures 4B–D**, the expression of MCT-1 and SMCT-1 was reduced in the KD mouse model, which was further decreased by antibiotics administration, maybe leading to a lower SCFAs absorption, and was partially recovered by *C. butyricum* supplement.

### Butyrate mitigated the inflammation in CAWS-treated RAW264.7 and mice

**Figure 5:**
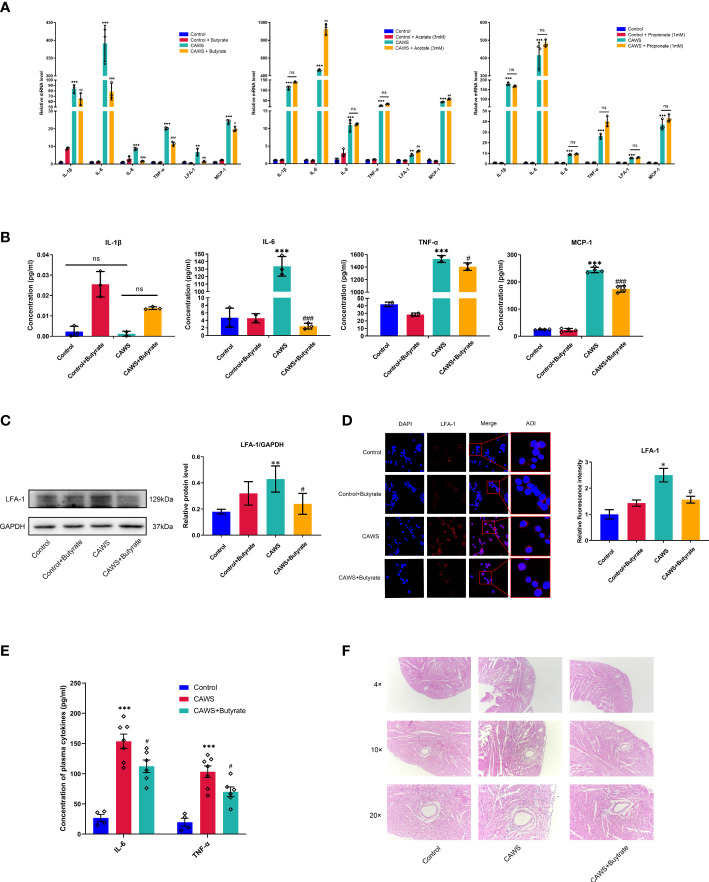
Butyrate mitigated the inflammation in CAWS-treated RAW264.7. **(A)** The mRNA expression of inflammation markers IL-1β, IL-6, IL-8, TNF-α, LFA-1 and MCP-1 was examined in RAW264.7 cells treated with acetate, propionate or butyrate. **(B)** The concentration of IL-1β, IL-6, TNF-α and MCP-1 was evaluated in the supernatant from RAW264.7 treated with butyrate by using ELISA. **(C)** The expression of LFA-1 was determined in RAW264.7 treated with butyrate by using western blot. **(D)** Relative fluorescence intensity was evaluated using immunofluorescent assay for LFA-1 in RAW264.7. Magnification: × 200. Scale bars = 10 µm. **(E)** Plasma cytokine levels of IL-6 and TNF-α. **(F)** H&E staining of heart tissues. Magnification: ×200. Scale bar = 20 μm. Values are expressed as the mean ± SEM. Significance: * P < 0.05, ** P < 0.01, *** P < 0.001, vs. the control group; ^#^ P < 0.05, ^##^ P < 0.01, ^###^ P < 0.01 vs. the CAWS group; ns means no significance.

In order to determine the effects of SCFAs on the inflammation of KD, acetate, propionate and butyrate, as the main component parts of fecal SCFAs, were chosen to observe the effects on the CAWS-induced inflammation in the mice-derived macrophages RAW264.7 cells. From **Figures 5A, B**, butyrate significantly decreased the inflammation-related markers, including IL-1β, IL-6, IL-8, TNF-α, LFA-1 and MCP-1, superior to the acetate or propionate. In further studies, we obtained the significant anti-inflammatory effect of butyrate on KD-sera-stimulated human-derived THP-1 and PBMC cells with the reduced IL-1β, IL-6, IL-8, TNF-α, LFA-1 and MCP-1 (**Supplementary Figure 2**). As a crucial chemokine and ligand for ICAM-1, LFA-1 serves as an important player in mediating the inflammation-related vascular injuries. In **Figures 5C, D**, CAWS stimulated the expression of LFA-1 in RAW264.7 cells, suggesting the importance of macrophages in inflammation-related vascular injuries, which was significantly downregulated by butyrate. Moreover, in view of the anti-inflammatory effect of butyrate *in vitro*, we have performed an experiment adding butyrate to the drinking water for CAWS-treated mice, and it also shown the therapeutic effect of butyrate (**Figures 5E, F**). It demonstrated that butyrate reduced the inflammatory cytokines IL-6 and TNF-α, and ameliorated the coronary lesions featured by decreased infiltration of inflammatory cells. Seen above, butyrate shows superior anti-inflammatory effect than acetate and propionate in macrophages of KD models.

### Butyrate promoted phosphatase MPK-1 expression to inhibit the MAPK signaling pathways in CAWS-stimulated RAW264.7

**Figure 6:**
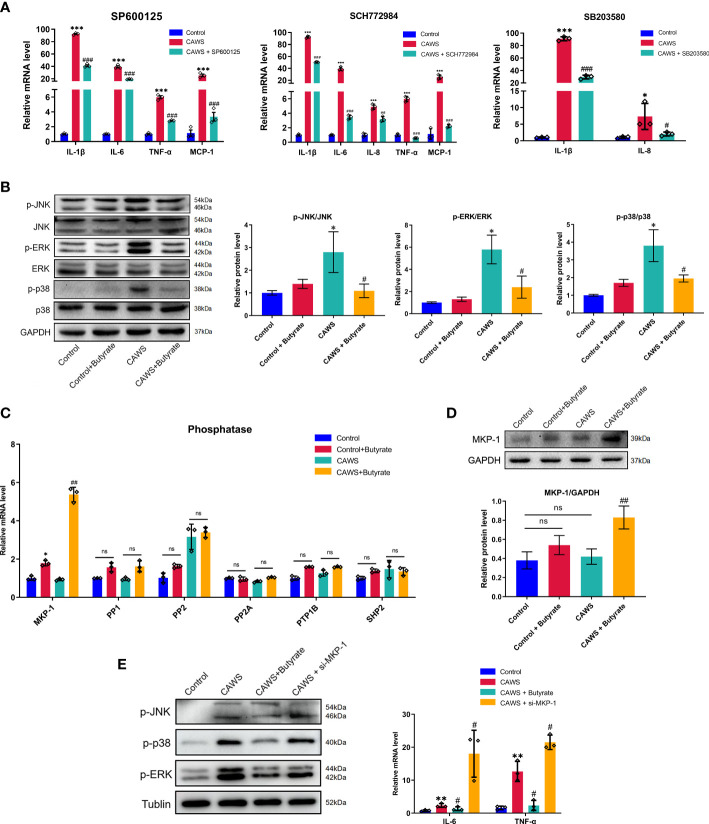
Butyrate promoted MPK-1 expression to inhibit the MAPK signaling pathways in CAWS-stimulated RAW264.7. **(A)** The mRNA expression level of IL-1β, IL-6, TNF-α and MCP-1 was examined in RAW264.7 cells treated with SP600125 (JNK inhibitor, 5 μmol/L), SCH772984 (ERK1/2 inhibitor, 300 nmol/L) and SB203580 (p38 inhibitor, 5 μmol/L), respectively. **(B)** The expression of phosphorylation of JNK, p38 and ERK was detected after butyrate treatment. **(C)** The mRNA expression of phosphatases MKP-1, PP1, PP2, PP2A, PTP1B and SHP2 in RAW264.7 after butyrate treatment. **(D)** The expression of MKP-1 was evaluated by using western blot. **(E)** The effect of MKP-1 knockdown was assessed by analyzing the phosphorylation level of JNK, p38 and ERK and expression of IL-6 and TNF-α. Values are expressed as the mean ± SEM. Significance: * P < 0.05, ** P < 0.01, *** P < 0.001, vs. the control group; ^#^ P < 0.05, ^##^ P < 0.01, ^###^ P < 0.01 vs. the CAWS group; ns means no significance.

In studies to date, JNK, ERK1/2, and p38 MAPK signaling pathways are suggested in initiating inflammatory reactions in KD (19, 20). Thus, we first used SP600125, SCH772984, and SB20358 which are inhibitors to JNK, ERK1/2 and p38, respectively, in CAWS-stimulated RAWS264.7 cells to validate the inflammatory role of them in KD (21). Hence, we examined a series of inflammatory cytokines including IL-1β, IL-6, IL-8, TNF-α, and MCP-1, which have been found significantly increased in KD mice. From **Figure 6A**, it suggested these kinase inhibitors significantly reduced the expression of different inflammatory markers, which implied the participation of JNK, ERK1/2 and p38 MAPK signaling pathways in KD. Since butyrate significantly mitigated the inflammation in CAWS-stimulated RAW264.7, we next investigated whether butyrate could act on the MAPK signaling pathways to fulfill its protective effect on RAWS264.7 cells. In **Figure 6B**, butyrate inhibited the activation of JNK, ERK1/2 and p38 in CAWS-stimulated RAW264.7 cells. Therefore, we speculated whether its protective effect could be related to the expression of phosphoprotein phosphatases (PPs) to dephosphorylate activated JNK, ERK1/2 and p38. Thus, among the known MPK-1, PP1, PP2, PP2A, PTP1B, and SHP2 detection for MAPK pathways inhibition, butyrate enhanced the MPK-1 expression in the CAWS-stimulated RAW264.7 cells, while no significant difference was observed in other PPs (**Figures 6C, D**). Furthermore, MKP-1 knockdown was found to promote the phosphorylation of JNK, ERK1/2, and p38, and increase inflammatory cytokines IL-6 and TNF-α, even higher than that in the CAWS group (**Figure 6E**).

As seen above, our results suggested butyrate increased expression of phosphatase MKP-1 to dephosphorylate activated JNK, ERK1/2 and p38 MAPK, consequently decreasing the inflammation in CAWS-stimulated RAW264.7 cells.

## Discussion

Gut microbiota is well-known about serving as a living barrier to maintain the host homeostasis and interact with the external environment (22, 23). However, in the first three years of life, gut microbiota at the initial stage is easily disrupted and participates in the pathogenesis of different diseases (24, 25). Interestingly, we observed the initial formation stage of gut microbiota largely overlaps with the age group of KD. In addition, a large proportion of KD patients experienced gastrointestinal symptoms including vomiting, diarrhea or constipation (5, 6), and altered gut microbiota composition was further shown during the acute phase of KD (26), whereas little is known about the details in the pathogenesis of KD. As current KD experimental protocols, extracts from intestine-colonized microorganisms *L. casei* and *C. albicans* are conventionally used to establish animal model of KD (7–9). Thus, it would be worthwhile to investigate the role of gut microbiota in KD.

The ratio of *Firmicutes* and *Bacteroidetes* (F/B) is widely used as a crucial barometer of the health condition, since the abundance *Bacteroidetes* is associated with inflammation and immune dysfunction and *Firmicutes* is related to the production of SCFAs (27). In the KD mouse model, we found the similar decreased F/B ratio of that from KD patients (8), with increased *Bacteroidetes* and reduced *Firmicutes*. In our findings, we concluded gut microbiota was crucial for regulation of inflammation status, when probiotic *C. butyricum* ameliorated inflammation level and vascular injuries, but gut dysbiosis aggravated by antibiotics promoted coronary neutrophils and macrophages recruitment and infiltration to worsen the inflammatory injuries. In some way, the protective effect could be achieved *via* its metabolites such as SCFAs. As one of the most bioactive microbial metabolites, SCFAs can be used locally by gut epithelial cells for maintenance of intestinal barrier or transported into circulation for parenteral anti-inflammation. A significant decrease in all detected fecal SCFAs were shown in KD mouse model and also reflected in a pilot study measuring fecal organic acid concentrations from KD patients, featured by significant decrease of butyrate (26). Moreover, recent experimental data demonstrated defective intestinal barrier with decreased expression of tight junction proteins such as Occludin, Claudin-3, and ZO-1 in LCWE-induced KD vasculitis model, and IVIG treatment could improve gut leakage to alleviate cardiovascular injuries (28). According to our findings, the reduced expression of Claudin-1, Jam-1, Occludin, and ZO-1 in the colon, was in line with increased plasma D-lactate, indicating a deficit in intestinal barrier function and increased gut permeability during the acute stage of KD. Taken together, the deficit of gut barrier function could result from the decline of fecal SCFAs for the reduced abundance of SCFAs-producing bacteria such as *Clostridium*, *Roseburia*, and *Bacteroides* in KD mouse model.

As a crucial part of the intestinal barrier, the gut microbiota is subject to many environmental factors, especially the use of antibiotics (29). Previous retrospective studies demonstrated the use of antibiotics can increase the risks of developing IVIG-resistant disease and have a lasting effect on the diversity and abundance of gut bacteria (30). Besides, antibiotics disrupt the Th17 and Treg differentiation and macrophage polarization by reducing SCFAs production, building a mechanistic link between gut dysbiosis and various inflammatory diseases. In our study, we demonstrated that the aggravated gut dysbiosis and disrupted intestinal barrier function caused by antibiotics could largely contribute to the higher level of inflammation and more severe vascular injuries in CAWS + ABX group, suggesting the health of gut microbiota was crucial for depressing the inflammatory status of the host, and urging for discontinuing unnecessary use of antibiotics.

Importantly, due to the anti-inflammatory effect of SCFAs on the hosts, we examined whether the transport of fecal SCFAs into circulation will be influenced. In our study, the expression of SCFAs transporters MCT-1 and SMCT-1 was found lowered in intestinal epithelial cells, which indicated the decline of plasma SCFAs level leading to inability of anti-inflammation. Activated macrophages were well-documented as the key driver of vascular inflammation in KD by piled studies (1, 31, 32). In our study, we chose the mice-derived RAW264.7 macrophages to investigate the anti-inflammatory mechanism of SCFAs. Our findings first demonstrated butyrate reduced inflammatory cytokines superior to other major fecal SCFA acetate and propionate, and also significantly inhibited the activation of JNK, ERK1/2 and p38 MAPK pathways in KD model. Since a series of phosphatases such as PP1, PP2A, PP2, PTP1B, SHP2, and MKP-1 were reported to target MAPK JNK, ERK and p38, we made further efforts to investigate whether butyrate exhibits its protective effects to inhibit all three MAPK pathways *via* modulating expression of certain phosphatases. Consequently, we only observed the increased expression of MPK-1 after butyrate treatment, while no significant difference was suggested from other PP candidates. In other studies of inflammatory diseases, MPK-1 activation could potentially contribute to the epithelial repair and its knockout would lead to upgraded inflammation and damages (33–36).

Apart from MAPK pathways, SCFA receptors activation could be another potential anti-inflammation mechanism (11, 37–39). GPR41 and GPR43 are a pair of G protein-coupled receptors (GPCRs), expressed in colon epithelial cells and peripheral blood mononuclear cells, and specifically activated by SCFAs to mediate interaction between gut microbiota and their host (38). GPR109A and Olfactory receptor-78 (Olfr-78) are another GPCRs, which are suggested with potential activity to bind with butyrate (40, 41). When knocking out these receptors, it may induce a series of inflammatory disorders such as colitis, arthritis or intraepithelial neoplasia (38). In our studies, we examined the expression of these receptors, and found increased expression of GPR109A in CAWS group and promoted expression of GPR41 after butyrate treatment (**Supplementary Figure 3**). However, due to the low activation of butyrate to GPR41, we do not consider the involvement of SCFA receptors activation as a major molecular mechanism of butyrate in KD. Furthermore, as a histone deacetylase inhibitor (HDACi), butyrate could enhance histone H3 acetylation to inhibit inflammation (42–45), and it would be interesting to explore the link between the increased MPK-1 and its HDACi effect in KD.

**Figure 7:**
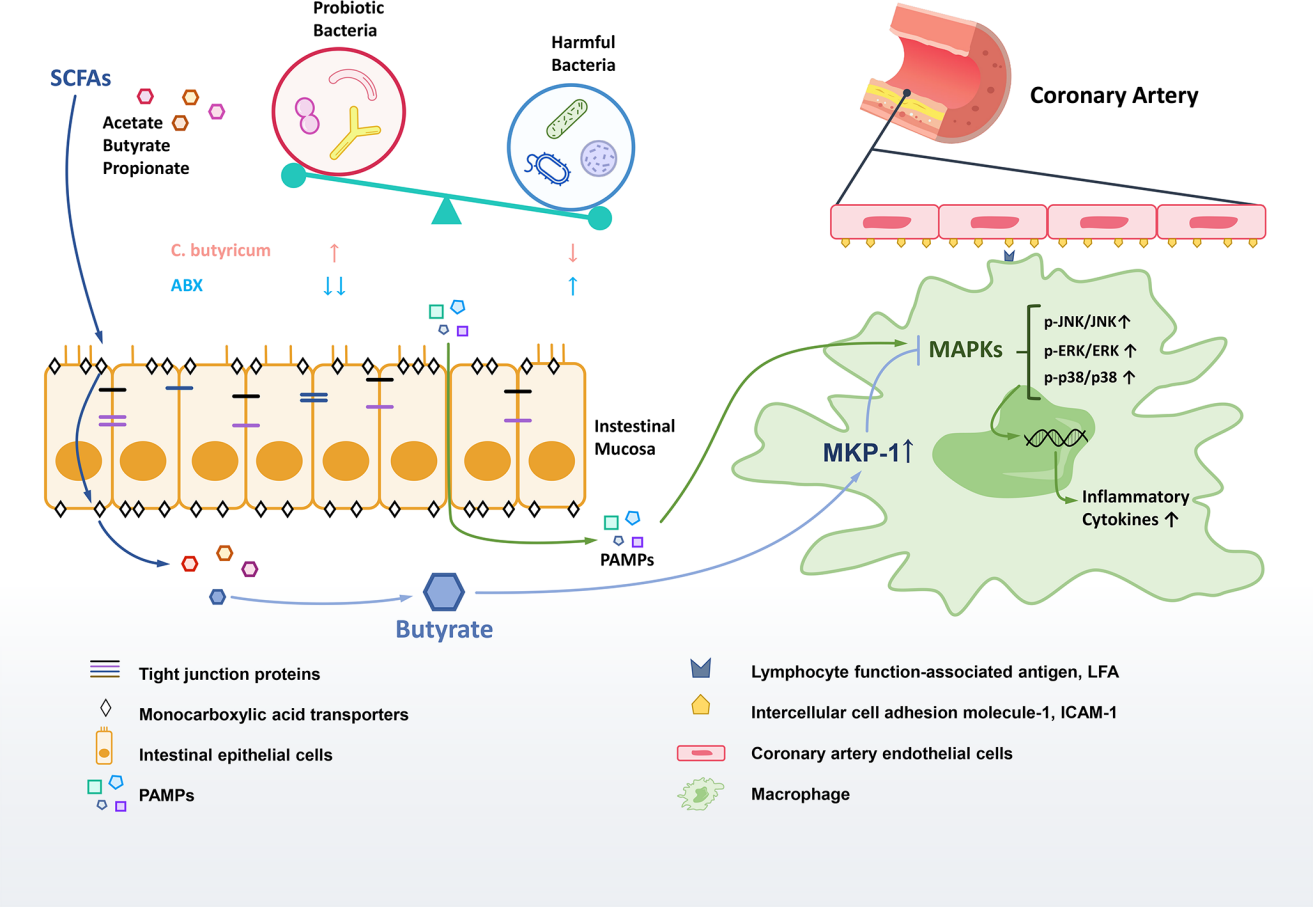
Gut dysbiosis in the pathogenesis of KD. The diversity of gut microbiota in the KD mouse model were reduced, especially the SCFAs-producing probiotics. The leaky gut was associated with the decrease of tight junction proteins in gut dysbiosis. The altered gut microbiota could influence on the production and transportation of SCFAs to regulate inflammatory responses. The potential therapeutic effect of butyrate may work on promoting the MKP-1 expression to inhibit MAPK pathway so as to reduce the inflammation.

Overall, we found that the reduction of SCFAs-producing bacteria and their metabolites SCFAs is the critical link between gut dysbiosis and inflammation in KD. The probiotic modification of gut microbiota with the increase of fecal SCFAs production in a murine model of KD was found to improve intestinal barrier, and significantly decrease the inflammation to ameliorate the coronary injuries. SCFA butyrate was exhibited significant anti-inflammation effects *via* increasing the expression of phosphatase MKP-1 to dephosphorylate activated JNK, ERK1/2 and p38 MAPK in RAW264.7 macrophages. Additionally, our results implied the use of antibiotics during the acute stage aggravated dysbiosis to aggravate the inflammation of KD (**Figure 7**).

## Data availability statement

The original contributions presented in the study are publicly available. This data can be found here: 10.6084/m9.figshare.23389202 (figshare).

## Ethics statement

The animal study was reviewed and approved by the Animal Care and Use Committee of Wenzhou Medical University.

## Author contributions

Conception and Design of study: FW, FQ, and QZ; Acquisition of data: FQ, QZ, and JHZ; Interpretation of data: JZ, JC, JMZ, and ML; Drafting the manuscript: FQ; Review and Editing: FW, QZ, and CJ; Supervision: FW and CJ; Project administration: XR and MC. All authors contributed to the article and approved the submitted version.

## Supplementary material

Supplementary Figure 1 Quantitative analyses of gut microbiota composition in mice. Venn diagram displayed significant quantitative differences of gut microbiota between groups.

Supplementary Figure 2 Anti-inflammatory effects of butyrate in THP-1 and PBMC. mRNA levels of IL-1β, IL-6, IL-8, TNF-α, LFA-1 and MCP-1 in THP-1 and PBMC cells among the control group, CAWS group and CAWS + butyrate group. Values are expressed as the mean ± SEM. Significance: * P < 0.05, ** P <0.01, *** P < 0.001, vs. the control group; ^##^ P < 0.01, ^###^ P < 0.01 vs. the CAWS group; ns means no significance.

Supplementary Figure 3 Effects of butyrate on SCFAs receptors. Relative mRNA levels of GPR109A, GPR43, GPR41 and Olfr78. Values are expressed as the mean ± SEM. Significance: *** P < 0.001, vs. the control group; ^##^ P < 0.01 vs. the CAWS group; ns means no significance.
