# Supplementary figures and images for: The reduced SCFA-producing gut microbes are involved in the inflammatory activation in Kawasaki disease
Source: Front Immunol. 2023 Jun 15;14:1124118. doi: 10.3389/fimmu.2023.1124118 (PMC10309029; doi:10.3389/fimmu.2023.1124118)

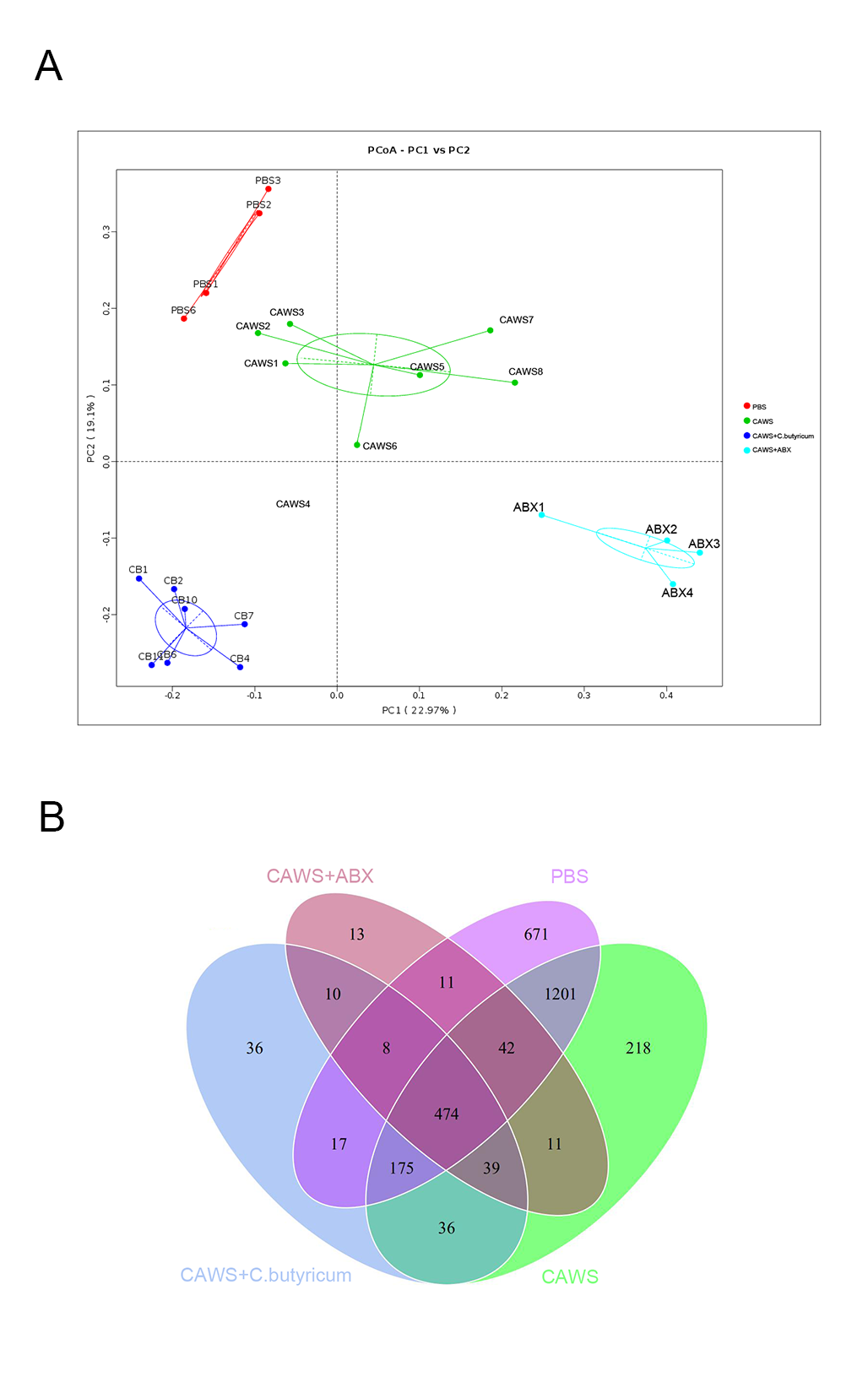

Supplement: Supplementary Figure 1 — Quantitative analyses of gut microbiota composition in mice. Venn diagram displayed significant quantitative differences of gut microbiota between groups. [file Image_1.tif]

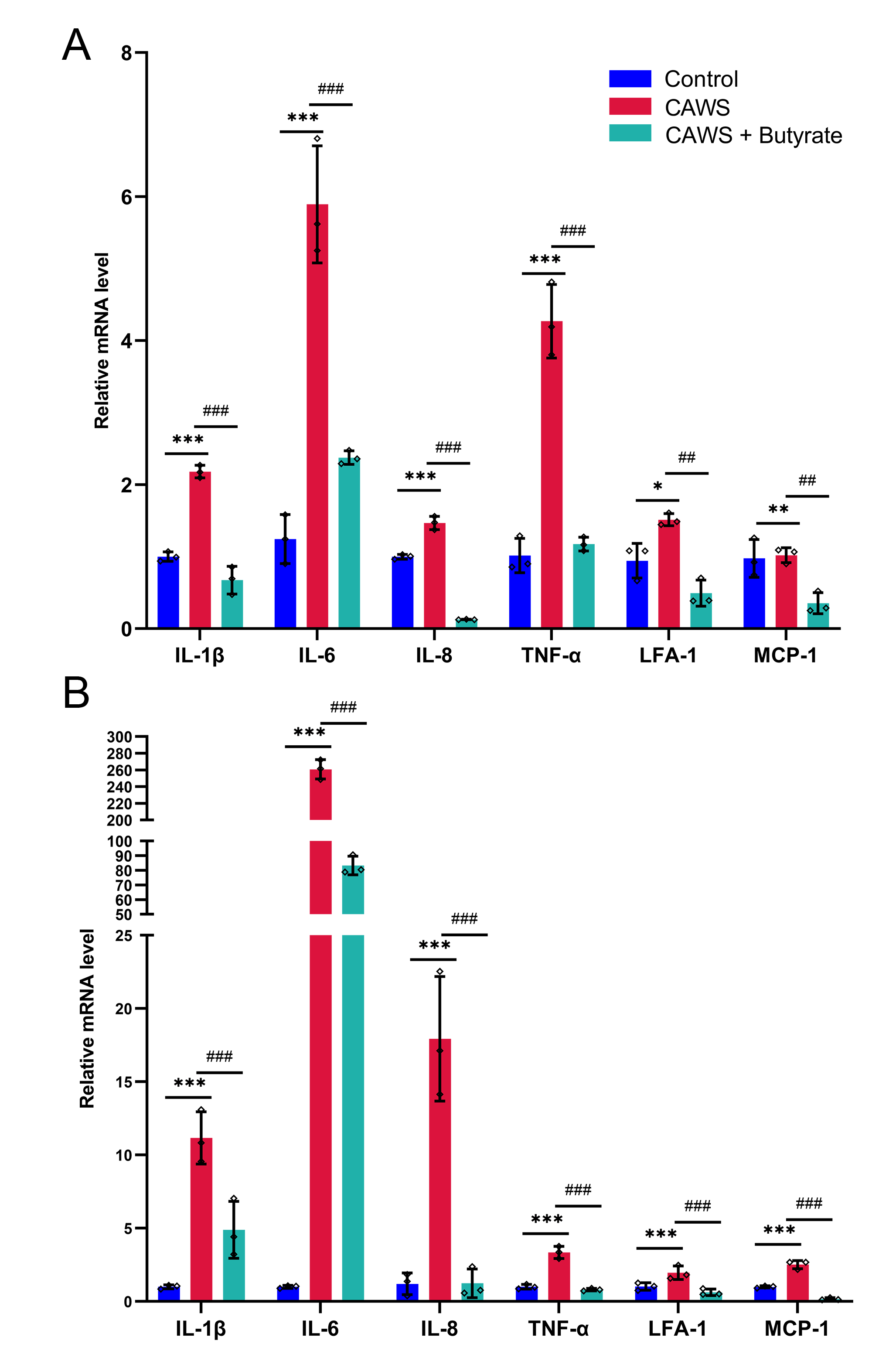

Supplement: Supplementary Figure 2 — Anti-inflammatory effects of butyrate in THP-1 and PBMC. mRNA levels of IL-1β, IL-6, IL-8, TNF-α, LFA-1 and MCP-1 in THP-1 and PBMC cells among the control group, CAWS group and CAWS + butyrate group. Values are expressed as the mean ± SEM. Significance: * P < 0.05, ** P <0.01, *** P < 0.001, vs. the control group; ## P < 0.01, ### P < 0.01 vs. the CAWS group; ns means no significance. [file Image_2.tif]

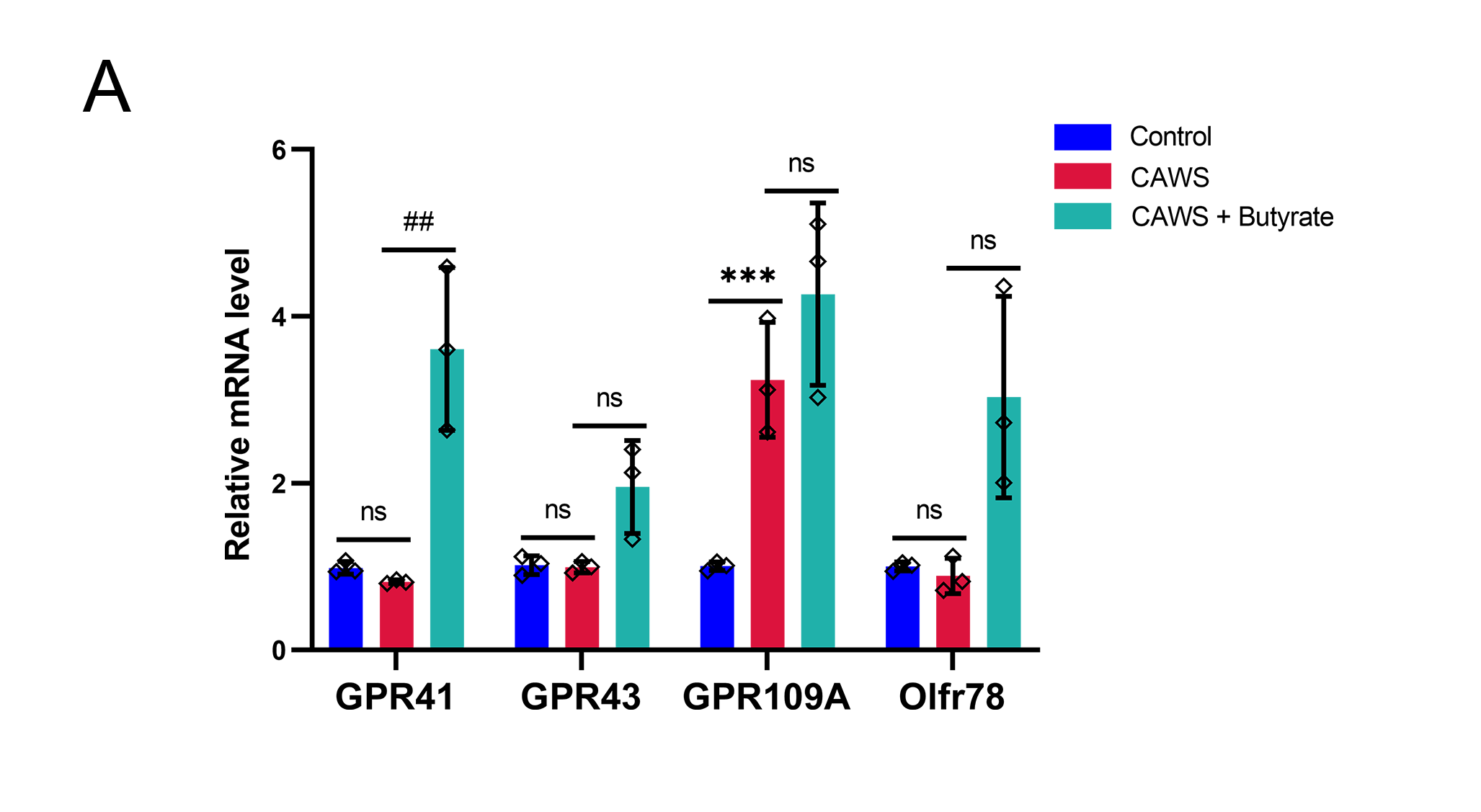

Supplement: Supplementary Figure 3 — Effects of butyrate on SCFAs receptors. Relative mRNA levels of GPR109A, GPR43, GPR41 and Olfr78. Values are expressed as the mean ± SEM. Significance: *** P < 0.001, vs. the control group; ## P < 0.01 vs. the CAWS group; ns means no significance. [file Image_3.tif]
