# Supplementary material for: The reduced SCFA-producing gut microbes are involved in the inflammatory activation in Kawasaki disease
Source: Front Immunol. 2023 Jun 15;14:1124118. doi: 10.3389/fimmu.2023.1124118 (PMC10309029; doi:10.3389/fimmu.2023.1124118)
Supplement: Supplementary file 4 [file Table_1.docx]

**Supplementary Table1. The primer details used in RT-qPCR**

| **Primers** | **Forward primer** | **Reverse primer** |
| --- | --- | --- |
| Claudin-1 | GGAGTCAGTGTTTCAGCCTATGGT | GAAGGGTTCATGCCTCTCATCT |
| Jam-1 | ATCCACCTCACTGTGCTTG | TTCTTGGCATCTGCTGTAAG |
| Occludin-1 | CCCAGATTAGAGTCCAAAGTCAGT | CGGAAACCTTAGAGAGATGCC |
| ZO-1 | AACCCGAAACTGATGCTGTG | CCCTTGGAATGTATGTGGAGAG |
| MCT-1 | GTGCTGACCCCAAGAAGGAATG | TGAGGTGGTTGTGGAAAAGGTAGTG |
| SMCT-1 | CCCTGGACATCTAAGAAAGTG | AGGCATTGATACTGGAGGAC |
| IL-1β | GAAATGATGGCTTATTACAGTGGCA | GTAGTGGTGGTCGGAGATTCGTAG |
| IL-6 | TCACAGAAGGAGTGGCTAAGGACC | ACGCACTAGGTTTGCCGAGTAGAT |
| IL-8 | TGTTCACAGGTGACTGCTCC | AGCCCATAGTGGAGTGGGAT |
| TNF-α | CTTGTTGCCTCCTCTTTTGCTTA | CTTTATTTCTCTCAATGACCCGTAG |
| LFA-1 | TGCTGACCAATACCTTTCG | GATGATGTAGCGGGTTATGTC |
| MCP-1 | GTGCTGACCCCAAGAAGGAATG | GTGCTGACCCCAAGAAGGAATG |
| MKP-1 | TGGTTCAACGAGGCTATTG | GCTTCACAAACTCAAAGGC |
| PP1 | TGGTTCAACGAGGCTATTG | CTCTGCTTCACAAACTCAAAGG |
| PP2 | ACTTGGAATCACGGGTTG | CCATCATTGTCTTTGTGTGG |
| PP2A | CACACAGGTTTATGGGTTCTAC | CAAGGCAGTGAGAGGAAGATAG |
| PTP1B | CACTGAAGTTAGGAGACGGATG | GCCAATGTGCCTTGTGTTC |
| SHP2 | GGACAGGAACCTTCATTGTG | GCAGCGTCTCTATGTAGTGC |
| GAPDH | AAGAAGGTGGTGAAGCAGG | GAAGGTGGAAGAGTGGGAGT |
